# Characterization of four mitochondrial genomes of family Neritidae (Gastropoda: Neritimorpha) and insight into its phylogenetic relationships

**DOI:** 10.1038/s41598-021-91313-0

**Published:** 2021-06-03

**Authors:** Jian-tong Feng, Li-ping Xia, Cheng-rui Yan, Jing Miao, Ying-ying Ye, Ji-ji Li, Bao-ying Guo, Zhen-ming Lü

**Affiliations:** 1 National Engineering Research Center for Marine Aquaculture, Zhejiang Ocean University, Zhoushan, 316022 China; 2 National Engineering Laboratory of Marine Germplasm Resources Exploration and Utilization, Zhejiang Ocean University, Zhoushan, 316022 China

**Keywords:** Evolution, Molecular biology

## Abstract

Neritidae is one of the most diverse families of Neritimorpha and possesses euryhaline properties. Members of this family usually live on tropical and subtropical coasts and are mainly gregarious. The phylogenetic relationships between several subclasses of Gastropoda have been controversial for many years. With an increase in the number of described species of Neritidae, the knowledge of the evolutionary relationships in this family has improved. In the present study, we sequenced four complete mitochondrial genomes from two genera (*Clithon* and *Nerita*) and compared them with available complete mitochondrial genomes of Neritidae. Gene order exhibited a highly conserved pattern among three genera in the Neritidae family. Our results improved the phylogenetic resolution within Neritidae, and more comprehensive taxonomic sampling of subclass Neritimorpha was proposed. Furthermore, we reconstructed the divergence among the main lineages of 19 Neritimorpha taxa under an uncorrelated relaxed molecular clock.

## Introduction

The mitochondrial genome (mitogenome) is typically circular in invertebrates and generally approximately 15–20 kb in size^1^. It usually contains 37 genes, divided into one control region, 13 protein-coding genes, two rRNA genes, and 22 tRNA genes, in which the number of tRNA genes is highly variable^2^. Due to rapid evolution, cellular abundance, and an absence of introns, mitochondrial sequences can be easily amplified. In addition, they have a compact size, maternal inheritance, conserved features in their gene organization, a lack of extensive recombination, and a higher mutation rate than nuclear sequences^3–5^. These sequences been extensively used in comparative and evolutionary genomics^6^, species identification, population genetics^7^, molecular evolutionary and phylogenetic analyses and taxonomic diagnosis in marine biological studies^8–10^. In particular, phylogenetic analysis based on complete mitogenomes proved that the resolution of inferred phylogenetic trees was improved compared with that of trees based on partial gene fragments^11^. With the rapid development of sequencing and amplification technology for complete mitogenomes, they have been widely used to reconstruct phylogenetic relationships in different gastropod groups^12–14^.

Gastropods from family Neritidae (Rafinesque, 1815) are the most diverse species of Neritimorpha. They are euryhaline, meaning that they occur in marine, brackish, and freshwater systems^15,16^. Members of this family live on tropical and subtropical coasts and usually inhabit the middle to upper intertidal rocky zones^17^. Neritidae graze on algae on rock surfaces^18,19^. This family is ecologically important in freshwater and marine ecosystems because it manages the growth of certain algae and acts as a food source for other organisms. Family Neritidae includes marine genera such as *Nerita*, whereas species from *Clithon* and *Neritina* prefer to inhabit estuaries, mangrove streams and intertidal muddy sand banks^15,16,20^. Species in the genus *Clithon* are common in brackish estuarine areas with little tidal influence^15^ and are often used to study morphological patterns due to their shell color and pattern variations^21–23^. The genus *Nerita* is the most prominent intertidal group along tropical shores. It is relatively abundant in the fossil record, first appearing in the Cretaceous. In addition, *Nerita* species display extensive dispersal potential, producing veliger larvae that stay in the plankton stage for weeks to months^24^.

This family and the whole subclass of Neritimorpha are unique branches of marine gastropods in terms of morphology, structure and phylogeny, and international research on these species is ongoing. There is a long history of taxonomic studies on family Neritidae. In 1815, Rafinesque formally established Neritidae at the rank of family^25^. In addition, synonyms caused by the classification of shell shape often occur, such as *Neritina zebra*, which was initially defined as a species of *Nerita* by Bruguière but was classified as a species of *Neritina* in later studies^26,27^. Subsequently, Haynes identified the genus by studying the differences between male and female reproductive systems in *Clithon* and *Neritina*^28^. Currently, with the development of molecular biology technology, mitogenome sequencing analysis is being increasingly applied in the phylogenetic analysis of family Neritidae. Moises et al. reconstructed the phylogeny of three species of snails by comparing their mitogenome sequences with those of other gastropods^29^. Feng et al. carried out sequence analysis, phylogenetic reanalysis and divergence time estimation of *Nerita undata* and *Nerita balteata* and eight other species of neritids^30^. To date, more than 14 entire Neritidae mitogenomes have been sequenced (https://www.ncbi.nlm.nih.gov). However, two-thirds of them belong to the genus *Nerita*; currently, only one complete mitogenome dataset is available for the genus *Clithon*.

In the present study, two new sequences of *Nerita* were obtained, and two sequences of *Clithon* were also provided, which will further clarify the phylogenetic relationships among different genera and even within the whole Neritimorpha subclass. We determined the complete mitogenomes of four Neritidae species, namely, *Clithon oualaniense* (Lesson 1831), *Clithon sowerbianum* (Récluz, 1843), *Nerita chamaeleon* (Linnaeus, 1758) and *Nerita japonica* (Dunker, 1860), which are widely distributed in the southeastern China Sea. The characteristics of the species were compared, and we evaluated the variation in and conservation of mitogenomes among Neritidae species. To better understand the functions of related genes, we analyzed the relative synonymous codon usage (RSCU) and AT skew values of protein coding genes (PCGs). Furthermore, the phylogeny of subclass Neritimorpha and related species was reconstructed, and the relationships between these taxa were discussed. The divergence time of four species in subclass Neritimorpha was evaluated, and selective pressure analysis was performed.

## Results and discussion

### Genome structure, organization, and composition

**Figure 1:**
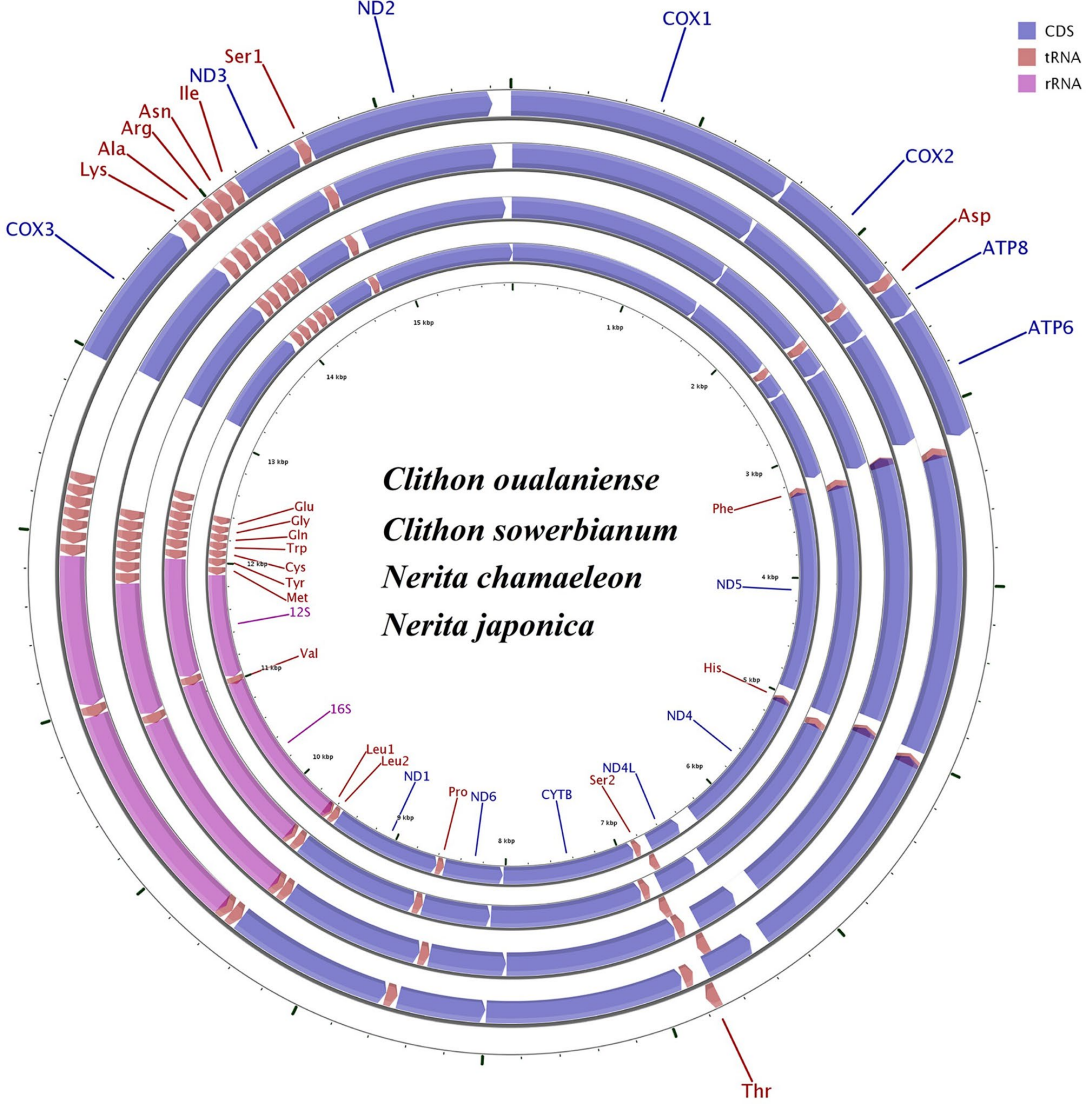
Gene map of the complete mitogenomes of *Clithon oualaniense* (GenBank accession No. MT568501), *Clithon sowerbianum* (MT230542), *Nerita chamaeleon* (MT161611) and *Nerita japonica* (MN747116). The ring indicates the gene arrangement and distribution. The largest ring is for *C. oualaniense,* and the smallest ring is for *N. japonica*. *ND1-6* NADH dehydrogenase subunits 1–6, *COX1-3* cytochrome c oxidase subunits 1–3, *ATP6 and ATP8* ATPase subunits 6 and 8, *CYTB* cytochrome b, *rRNA* ribosomal RNA gene, *tRNA* transfer RNA gene.

The entire mitogenome sequences of the four Neritimorpha species have lengths of 15,706 bp for *C. oualaniense*, 15,919 bp for *C. sowerbianum*, 15,716 bp for *N. chamaeleon* and 15,875 bp for *N. japonica* (GenBank accessions MT568501, MT230542, MT161611 and MN747116, respectively) (Table 1). The four circular molecules encode seven PCGs, eight tRNA genes on the forward strand, and 22 other mitochondrial genes on the reverse strand in the same orientation (Table 2). The control region is located between the *cox3* and *trnE* genes, similar to the pattern in other previous reports on Neritidae species^29–35^ (Fig. 1). The genome structures of the four species were identical to those of other Neritimorpha taxa, without gene rearrangement, which may be related to their life history and habitat.

**Table 1:** List of species analyzed in this study and their GenBank accession numbers.

Subclass	Family	Species	Size (bp)	Accession no
Vetigastropoda	Turbinidae	*Angaria delphinus*	19,554	NC_031860
		*Angaria neglecta*	19,470	NC_028707
		*Astralium haematragum*	16,310	NC_031858
		*Bolma rugosa*	17,432	NC_029366
		*Lunella aff. Cinereal*	17,670	KF700096
		*Lunella granulate*	17,190	NC_031857
	Tegulidae	*Tegula brunnea*	17,690	NC_016954
		*Tegula lividomaculata*	17,375	NC_029367
		*Tectus pyramis*	18,439	MF138911
	Trochidae	*Gibbula umbilicalis*	16,277	NC_035682
		*Stomatella planulata*	17,151	NC_031861
		*Umbonium thomasi*	15,998	MH729882
	Haliotidae	*Haliotis discus hannai*	16,886	KF724723
		*Haliotis rufescens*	16,646	NC_036928
		*Haliotis iris*	17,131	NC_031361
		*Haliotis laevigata*	16,545	NC_024562
		*Haliotis rubra*	16,907	AY588938
		*Haliotis tuberculata*	16,521	FJ599667
	Phasianellidae	*Phasianella solida*	16,698	NC_028709
Neomphaliones	Bathysciadiidae	*Bathysciadiidae sp.*	17,238	MH837532
	Cocculinidae	*Cocculina subcompressa*	18,167	MH837536
	Peltospiridae	*Peltospira smaragdina*	15,112	MH837538
Caenogastropoda	Muricidae	*Boreotrophon candelabrum*	15,265	NC_046505
		*Ceratostoma burnetti*	15,334	NC_046569
		*Ceratostoma rorifluum*	15,338	MK411750
		*Ocinebrellus falcatus*	15,326	NC_046052
		*Ocinebrellus inornatus*	15,324	NC_046577
		*Concholepas concholepas*	15,495	NC_017886
		*Rapana venosa*	15,272	EU170053
	Conidae	*Conus betulinus*	16,240	NC_039922
		*Conus tulipa*	15,756	KR006970
		*Conus borgesi*	15,536	EU827198
		*Conus capitaneus*	15,829	NC_030354
		*Conus tribblei*	15,570	NC027957
	Turridae	*Turricula nelliae spuria*	16,453	MK251986
	Naticidae	*Euspira gilva*	15,315	NC_046593
		*Euspira pila*	15,244	NC_046703
		*Mammilla kurodai*	15,309	NC_046596
		*Mammilla mammata*	15,319	NC_046597
	Xenophoridae	*Onustus exutus*	16,043	MK327366
	Pomatiopsidae	*Oncomelania hupensis nosophora*	15,182	LC276226
		*Oncomelania quadrasi*	15,184	LC276227
		*Oncomelania hupensis robertsoni*	15,188	LC276228
	Turritellidae	*Turritella bacillum*	15,868	NC_029717
	Epitoniidae	*Epitonium scalare*	15,143	MK251987
Neritimorpha	Neritidae	*Clithon oualaniense*	15,706	MT568501
		*Clithon retropictus*	15,802	NC_037238
		*Clithon sowerbianum*	15,919	MT230542
		*Neritina usnea* (partial genome)	15,574	KU342665
		*Neritina violacea*	15,710	KY021066
		*Nerita albicilla*	15,314	MK516738
		*Nerita balteata*	15,571	MN477253
		*Nerita chamaeleon*	15,716	MT161611
		*Nerita undata*	15,583	MN477254
		*Nerita versicolor*	15,866	KF728890
		*Nerita fulgurans*	15,343	KF728888
		*Nerita tessellata*	15,741	KF728889
		*Nerita japonica*	15,875	MN747116
		*Nerita yoldii*	15,719	MK395169
		*Nerita melanotragus*	15,261	GU810158
	Helicinidae	*Pleuropoma jana*	15,851	KU342666
Patellogastropoda	Acmaeidae	*Bathyacmaea nipponica*	16,792	MF095859
	Nacellidae	*Cellana radiata*	16,194	MH916651
		*Nacella clypeater*	16,742	KT990124
		*Nacella magellanica*	16,663	KT990125
		*Nacella concinna*	16,761	KT990126
	Patellidae	*Patella ferruginea*	14,400	MH916654
		*Patella vulgata*	14,808	MH916653
	Lottiidae	*Lottia digitalis*	26,835	DQ238599
		*Lottia goshimai*	18,192	MT248298
		*Nipponacmea fuscoviridis*	18,720	MK395167
Heterobranchia	Aplysiidae	*Aplysia californica*	14,117	AY569552
		*Aplysia dactylomela*	14,128	DQ991927
		*Aplysia kurodai*	14,131	KF148053
	Polyceridae	*Nembrotha kubaryana*	14,395	NC_034920
		*Roboastra europaea*	14,472	NC_004321
		*Notodoris gardineri*	14,424	DQ991934
	Siphonariidae	*Siphonaria pectinate*	14,065	AY345049
	Volvatellidae	*Ascobulla fragilis*	14,745	AY345022
	Placobranchidae	*Elysia cornigera*	14,118	NC_035489
		*Elysia timida*	14,088	NC_035490
	Ellobiidae	*Auriculastra duplicata*	13,920	NC_036959
		*Auriculinella bidentata*	14,135	JN606066
		*Ovatella vulcani*	14,274	JN615139
	Onchidiidae	*Onchidella celtica*	14,150	AY345048
		*Peronia peronii*	13,968	JN619346
		*Platevindex mortoni*	13,991	NC_013934
	Pyramidellidae	*Pyramidella dolabrata*	13,856	AY345054

**Table 2:** Summary of the gene features of *Clithon oualaniense*, *Clithon sowerbianum*, *Nerita chamaeleon* and *Nerita japonica*.

Gene	Strand	Size (bp)	Initiation codon	Termination codon	Intergenic nucleotide*(bp)	Anticodon
*cox1*	+	1548	ATG	TAA	11/11/5/5	
*cox2*	+	690	ATG	TAA/TAG	1/1/12/15	
*trnD*	+	66–67			0	GTC
*atp8*	+	165	ATG	TAA/TAG	5/6/10/10	
*atp6*	+	699–702	ATG	TAA/TAG	22/25/31/34	
*trnF*	−	66–70			− 29/− 60/− 29/− 29	GAA
*nad5*	−	1665–1717	ATT	TAA	27/57/57/78	
*trnH*	−	66–67			− 47/− 47/− 20/− 47	GTG
*nad4*	−	1254–1323	ATG	TAA	83/152/83/152	
*nad4l*	−	294	ATG	TAA	4	
*trnT*	+	68			5/8/3/3	TGT
*trnS2*	−	65			5	CGA
*cob*	−	1137	ATG	TAA	5/4/6/5	
*nad6*	−	501–507	ATG/ATT	TAA	7/1/1/1	
*trnP*	−	66			1	TGG
*nad1*	−	933	ATG	TAA/TAG	0	
*trnL2*	−	68			0/0/14/0	TAA
*trnL1*	−	57–71			− 25/− 25/− 27/− 19	TAG
*rrnL*	−	1318–1334			− 7/− 7/− 11/− 4	
*trnV*	−	67–68			− 1	TAC
*rrnS*	−	863–870			− 1/− 1/0/0	
*trnM*	−	67–68			4/4/7/5	CAT
*trnY*	−	68			4/4/1/2	GTA
*trnC*	−	64–66			0	GCA
*trnW*	−	66–69			0	TCA
*trnQ*	−	69			0/0/1/1	TTG
*trnG*	−	65–67			3/2/12/12	TCC
*trnE*	−	66			637/834/613/80	TTC
*cox3*	+	780	ATG	TAA/TAG	33/25/20/36	
*trnK*	+	67–68			20/19/7/8	TTT
*trnA*	+	68–69			11/13/15/14	TGC
*trnR*	+	69			2/13/6/12	TCG
*trnN*	+	72–74			4/8/2/6	GTT
*trnI*	+	69			0/1/0/0	GAT
*nad3*	+	354	ATG	TAA/TAG	3/3/8/5	
*trnS1*	+	68			0/0/57/0	GCT
*nad2*	+	1003–1101	ATG/ATT	T(AA)	99/99/42/1	

Intergenic Nucleotide*(bp): positive values indicated the interval sequence of adjacent genes, and negative values indicated the overlapping of adjacent genes.

The nucleotide compositions of the four whole mitogenomes were A: 29.81% to 33.79%, T: 30.67 to 35.36, G: 15.24 to 21.18, and C: 13.66 to 20.30 (Table 3). The contents of A and T exhibited high values, indicating codon usage bias towards A and T. The G and C contents of the four species were low, indicating an obvious bias against G and C. Moreover, the base compositions of 14 species in family Neritidae of the Neritimorpha were compared (Table 4). The AT contents of the 14 entire mitogenomes ranged from 61.67% to 66.28%, while the AT skew of most species was negative (− 0.1117 to − 0.0438), indicating the occurrence of fewer A than T nucleotides, except in *C. sowerbianum* (0.0484).

**Table 3:** Nucleotide composition of the mitogenomes of four Neritidae species.

Region	Size (bp)		A (%)		T (%)		G (%)		C (%)	
	Co	Cs	Co	Cs	Co	Cs	Co	Cs	Co	Cs
Mitogenome	15,706	15,919	31.46	33.79	34.34	30.67	19.11	15.24	15.08	20.30
*cox1*	1548	1548	23.51	22.55	40.44	39.66	21.19	22.22	14.86	15.57
*cox2*	690	690	27.25	27.25	36.38	36.38	21.01	21.16	15.36	15.22
*atp8*	165	165	29.09	28.48	40.61	42.42	19.39	19.39	10.91	9.70
*atp6*	702	699	23.08	22.46	41.74	41.06	19.52	20.46	15.67	16.02
*cox3*	780	780	21.41	20.77	40.51	39.23	22.18	23.97	15.90	16.03
*nad3*	354	354	21.75	18.64	43.22	43.50	23.73	26.27	11.02	11.58
*nad1*	933	933	27.76	26.05	37.41	36.76	16.29	17.15	18.54	20.04
*nad5*	1716	1717	28.96	28.65	35.96	33.84	14.28	14.85	20.80	22.66
*nad4*	1323	1254	27.97	27.43	38.10	37.16	14.36	14.59	19.58	20.81
*nad4l*	294	294	28.57	29.59	35.71	36.05	17.69	17.01	18.03	17.35
*nad6*	501	507	27.15	25.05	43.51	40.04	13.77	15.98	15.57	18.93
*cob*	1137	1137	26.47	26.47	37.03	36.94	15.57	15.30	20.93	21.28
*nad2*	1003	999	24.03	24.02	41.48	41.14	22.23	23.22	12.26	11.61
tRNAs	1481	1485	30.79	31.18	32.14	32.26	21.74	20.94	15.33	15.62
rRNAs	2193	2196	36.62	36.57	31.19	30.51	17.10	17.12	15.09	15.80
PCGs	11,146	11,077	25.96	25.30	38.90	38.02	18.01	18.78	17.12	17.89

Region	Size (bp)		A (%)		T (%)		G (%)		C (%)	
	Nc	Nj	Nc	Nj	Nc	Nj	Nc	Nj	Nc	Nj
Mitogenome	15,716	15,875	30.40	29.81	35.36	35.35	20.53	21.18	13.71	13.66
*cox1*	1548	1548	22.55	20.93	41.02	40.89	21.90	23.64	14.53	14.53
*cox2*	690	690	26.09	25.22	36.81	37.68	23.62	23.62	13.48	13.48
*atp8*	165	165	27.27	27.88	39.39	39.39	21.82	21.82	11.52	10.91
*atp6*	699	699	22.03	19.60	43.63	44.06	19.74	21.60	14.59	14.74
*cox3*	780	780	19.87	21.03	42.05	40.26	23.08	23.85	15.00	14.87
*nad3*	354	354	19.21	18.08	46.61	46.05	25.14	27.40	9.04	8.47
*nad1*	933	933	29.26	29.26	35.37	33.55	15.22	14.68	20.15	22.51
*nad5*	1686	1665	31.55	31.83	33.93	33.03	12.51	12.97	22.00	22.16
*nad4*	1296	1254	29.55	30.14	37.65	35.73	12.27	12.60	20.52	21.53
*nad4l*	294	294	33.67	32.65	32.65	33.33	14.29	15.31	19.39	18.71
*nad6*	507	507	30.37	29.19	40.04	40.04	11.44	13.02	18.15	17.75
*cob*	1137	1137	27.70	28.41	36.24	36.50	14.60	14.86	21.46	20.23
*nad2*	1003	1101	22.13	21.44	41.08	42.96	25.32	25.70	11.47	9.90
tRNAs	1485	1495	31.52	30.90	31.92	32.24	21.48	21.54	15.08	15.32
rRNAs	2204	2239	35.66	36.62	30.90	30.15	16.11	15.72	17.33	17.51
PCGs	11,092	11,127	26.41	25.82	38.45	38.73	17.82	19.31	17.32	16.14

**Table 4:** Summary of the base composition of the mitogenomes from 14 species in family Neritidae of the Neritimorpha.

Species (Neritidae)	Length (bp)	Entire Genome			Length (bp)	PCGs		
		AT%	AT-skew	GC-skew		AT%	AT-skew	GC-skew
*Nerita undata*	15,583	63.18	− 0.1010	0.2442	11,271	62.26	− 0.1928	0.0080
*Nerita balteata*	15,571	63.29	− 0.1019	0.2412	11,271	62.36	− 0.1953	0.0099
*Nerita albicilla*	15,314	64.49	− 0.0532	0.1639	10,875	64.01	− 0.0577	0.1914
*Nerita yoldii*	15,719	64.71	− 0.1117	0.0448	11,097	63.84	− 0.1830	0.0227
*Nerita fulgurans*	15,343	64.37	− 0.0679	0.1892	11,346	63.81	− 0.1909	0.0252
*Nerita tessellata*	15,741	64.05	− 0.0532	0.1771	11,337	63.21	− 0.1936	0.0242
*Nerita versicolor*	15,866	61.67	− 0.0650	0.1725	11,337	60.43	− 0.2014	0.0106
*Nerita melanotragus*	15,261	63.54	− 0.0680	0.1637	11,321	62.72	− 0.1799	0.0019
*Clithon retropictus*	15,802	64.87	− 0.0449	0.1500	11,283	64.03	− 0.2013	− 0.0014
*Clithon oualaniense*	15,706	65.80	− 0.0438	0.1181	11,146	64.86	− 0.1994	0.0253
*Clithon sowerbianum*	15,919	64.46	0.0484	− 0.1425	11,077	63.32	− 0.2009	0.0241
*Nerita chamaeleon*	15,716	65.76	− 0.0755	0.1992	11,092	64.86	− 0.1857	0.0144
*Nerita japonica*	15,875	65.16	− 0.0851	0.2161	11,127	64.55	− 0.2000	0.0896
*Neritina violacea*	15,710	66.28	− 0.0534	0.1548	11,312	65.64	− 0.1973	0.0047

Species (Neritidae)	Length (bp)	tRNAs			Length (bp)	rRNAs		
		AT%	AT-skew	GC-skew		AT%	AT-skew	GC-skew
*Nerita undata*	1497	62.53	− 0.0171	0.1800	2236	65.88	0.0957	− 0.0485
*Nerita balteata*	1497	62.86	− 0.0223	0.1583	2231	65.62	0.0929	− 0.0509
*Nerita albicilla*	1498	62.55	− 0.0309	0.0232	2243	66.39	0.0692	− 0.0159
*Nerita yoldii*	1428	63.79	− 0.0165	0.1682	2154	67.22	0.0925	− 0.0510
*Nerita fulgurans*	1510	63.58	− 0.0104	0.1637	2166	65.81	0.0869	− 0.0608
*Nerita tessellata*	1510	63.25	− 0.0199	0.1820	2165	66.11	0.0852	− 0.0424
*Nerita versicolor*	1513	62.06	− 0.0268	0.1603	2168	65.18	0.0913	− 0.0517
*Nerita melanotragus*	1426	63.35	− 0.0044	0.1607	2165	67.07	0.0743	− 0.0323
*Clithon retropictus*	1493	63.93	− 0.0142	0.1400	2160	67.04	0.0967	0.0197
*Clithon oualaniense*	1481	62.93	− 0.0215	0.1730	2193	67.81	0.0800	0.0623
*Clithon sowerbianum*	1485	63.44	− 0.0170	0.1455	2196	67.08	0.0903	0.0401
*Nerita chamaeleon*	1485	63.44	− 0.0064	0.1750	2204	66.56	0.0716	− 0.0366
*Nerita japonica*	1495	63.14	− 0.0212	0.1688	2239	66.77	0.0970	− 0.0538
*Neritina violacea*	1483	62.71	− 0.0365	0.1899	2164	67.74	0.0614	0.0372

### PCGs, tRNA genes, rRNA genes and codon usage

The AT contents of PCGs (− 0.2014 to − 0.0577) and tRNAs (− 0.0365 to − 0.0044) in the 14 Neritidae species had the same base bias as the entire genome (Table 4); however, the AT skew of the rRNAs (0.0614 to 0.0970) was slightly positive. All AT skew values were negative, while most GC skew values were positive. The AT content values of PCGs ranged from 60.43% to 65.64% in the 14 Neritidae species, indicating strong AT bias. All PCGs in the four mitogenomes started with the conventional initiation codon ATG or ATT and stopped with TAA or TAG.

**Figure 2:**
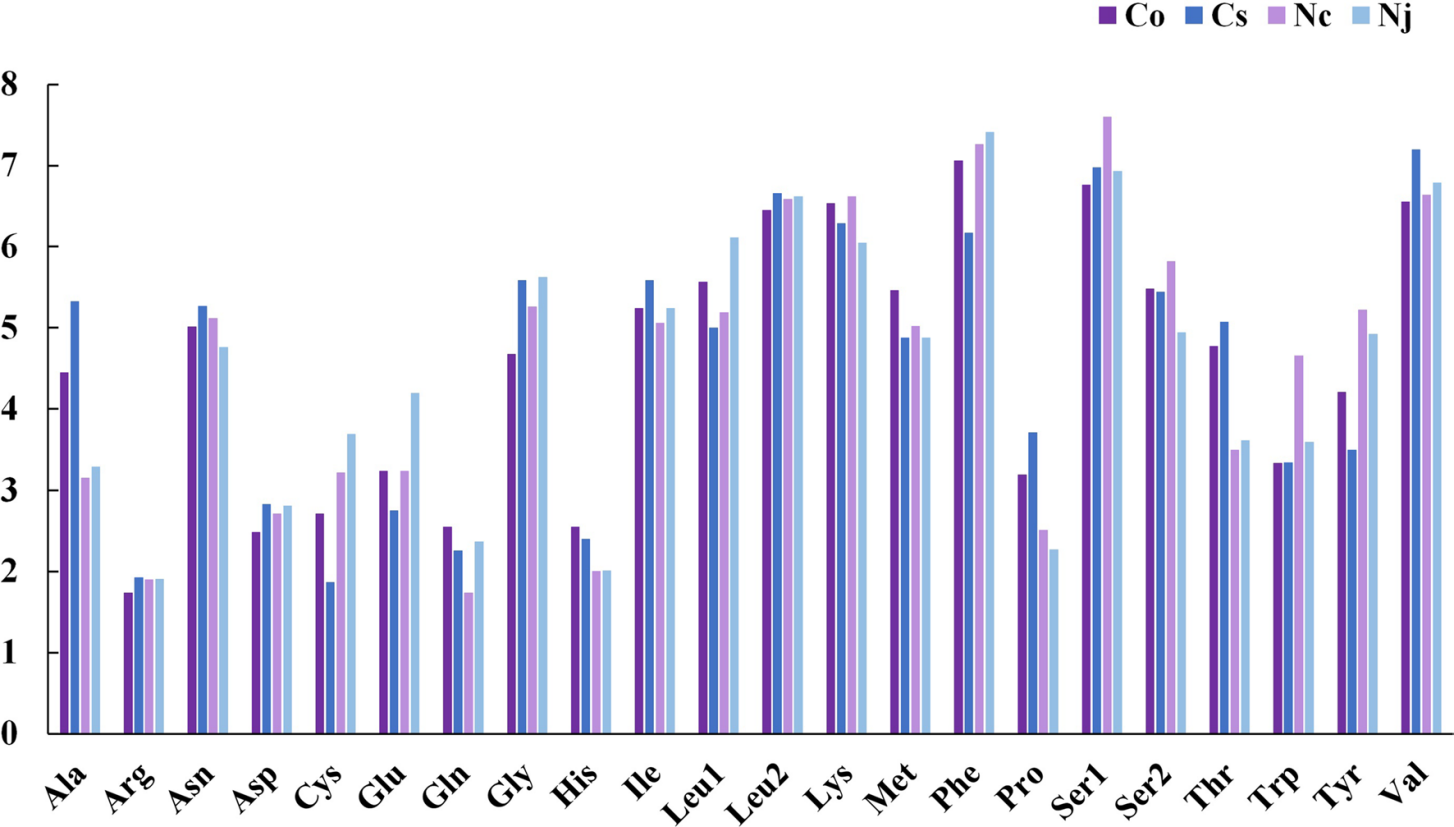
Percentage of each amino acid for proteins coded by PCGs in the four newly obtained mitochondrial genomes of *C. oualaniense*, *C. sowerbianum*, *N. chamaeleon*, and *N. japonica*.

**Figure 3:**
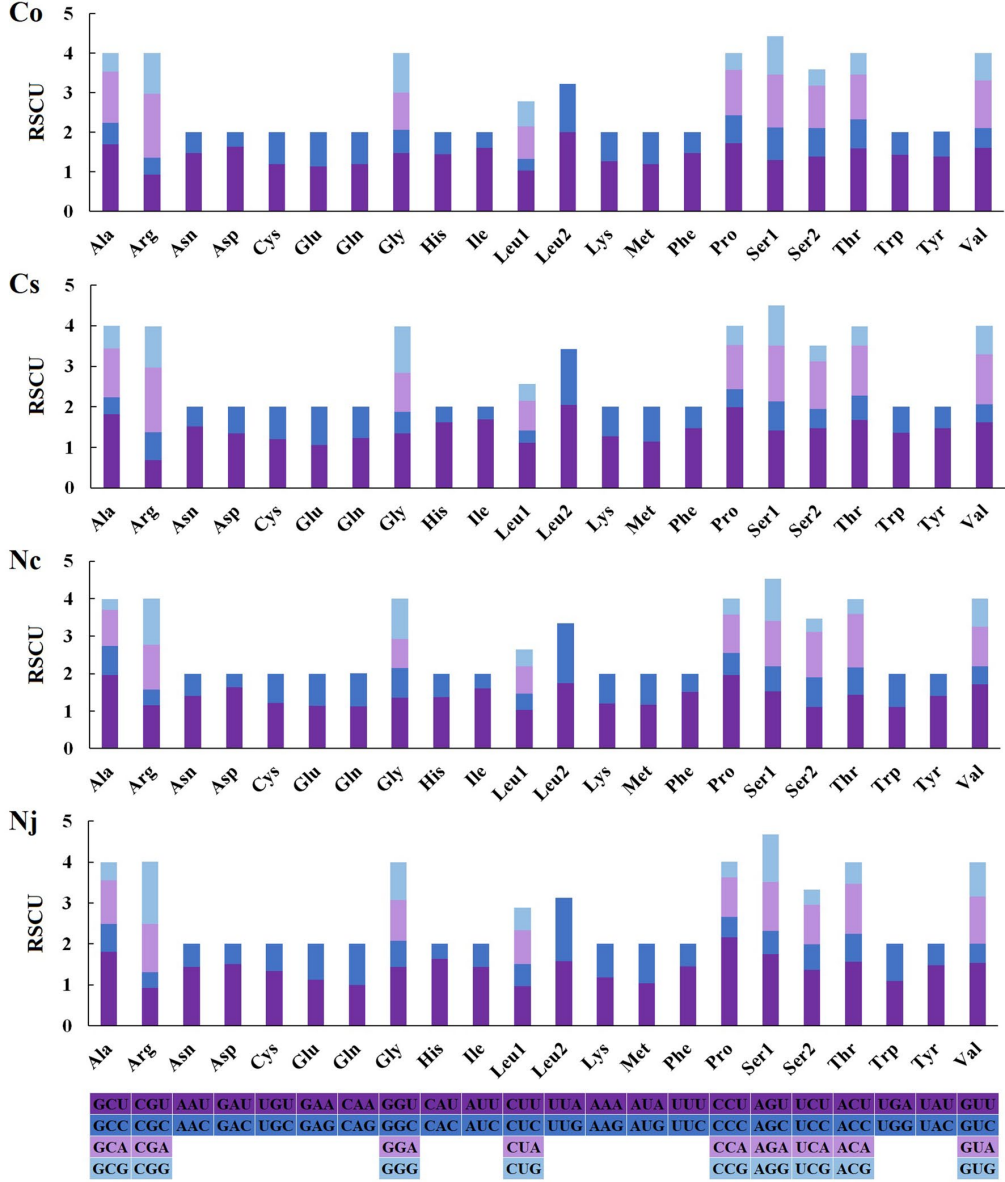
The relative synonymous codon usage (RSCU) in the mitochondrial genomes of four Neritidae species. Co indicates the RSCU of *C. oualaniense*, Cs indicates the RSCU of *C. sowerbianum*, Nc indicates the RSCU of *N. chamaeleon*, and Nj indicates the RSCU of *N. japonica*.

The most frequently utilized amino acids in the four species were *Leu2*, *Lys*, *Phe*, *Ser1* and *Val* (with frequencies ranging from 6.17% and 7.60%) (Fig. 2). The least common amino acid was *Arg* (all frequencies less than 2%), which is similar to the pattern previously reported in two Neritidae species (*N. undata* and *N. balteata*)^30^. Relative synonymous codon usage (RSCU) values for the 13 PCGs showed that UUA (*Leu2*) and CCU (*Pro*) were the two most frequent codons in the *Clithon* species (Fig. 3), and the most frequent codons in the *Nerita* species were CCU (*Pro*) and GCU (*Ala*). The 13 PCGs ranged in size from 165 bp (*atp8* of all Neritidae) to 1717 bp (*nad5* of *C. sowerbianum*). It is noteworthy that the *atp8* gene is the smallest PCG in all currently described neritids. These comparative analyses showed that codon usage patterns are conserved among Neritidae species.

The lengths of the tRNA genes were almost identical among the four Neritidae species, ranging from 57 (*trnL1* of *N. chamaeleon*) to 74 bp (*trnN* of two *Nerita* species). The AT contents of tRNA genes ranged from 62.06% to 63.93% in the 14 Neritidae species (Table 4). The *rrnL* genes of the four Neritidae species were 1318 to 1334 bp in length, while the *rrnS* genes were 863 to 870 bp. In general, the A and T contents were greater than the G and C contents in the two rRNA genes (Table 3).

### Selective pressure analysis

**Figure 4:**
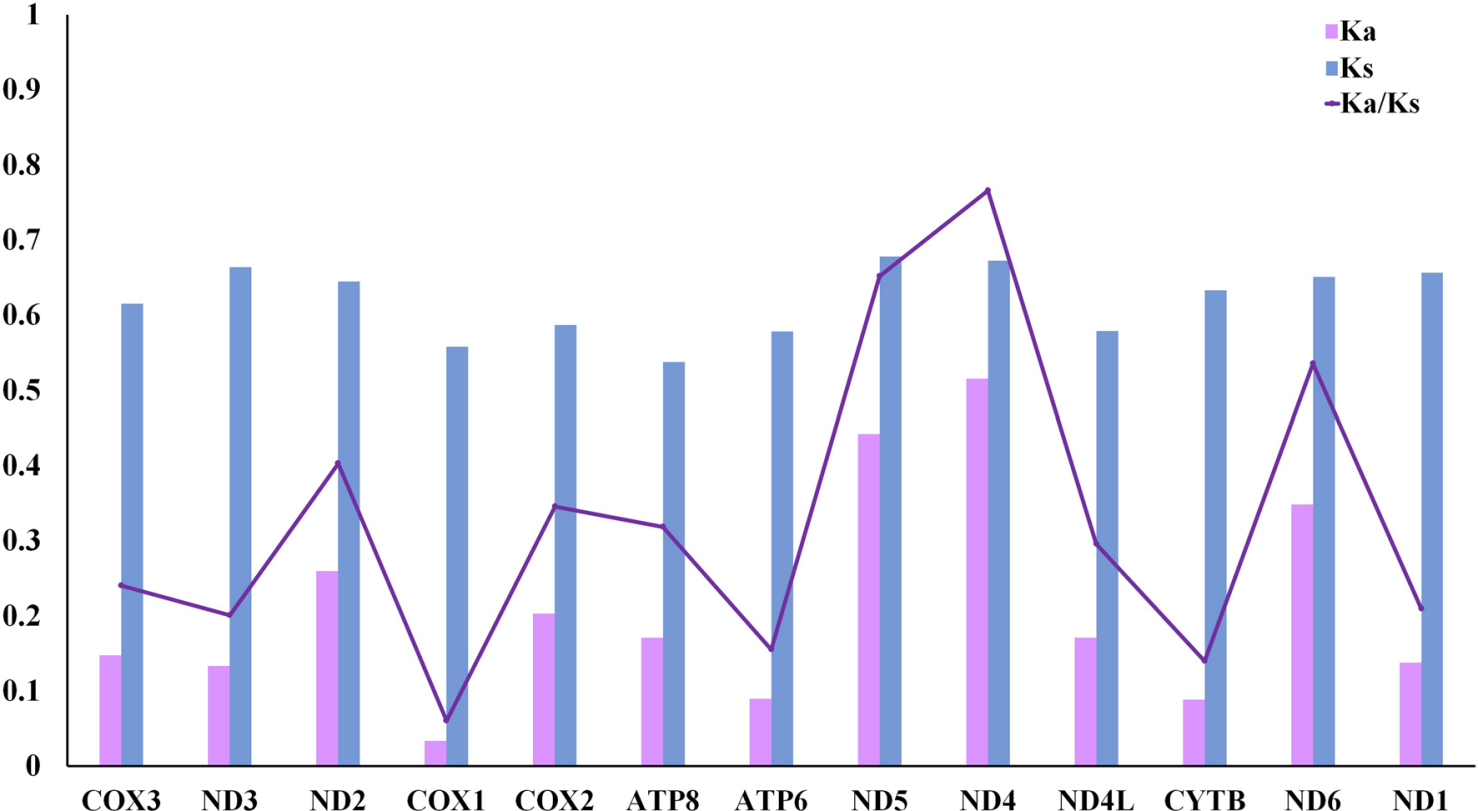
The purple line indicates the mean pairwise divergence of the Ka/Ks ratio for 13 PCGs among 16 Neritimorpha mitochondrial genomes. The 16 species of Neritimorpha are listed in Table 1. The pink and blue boxes indicate the number of nonsynonymous substitutions per nonsynonymous site (Ka) and the number of synonymous substitutions per synonymous site (Ks), respectively.

**Figure 5:**
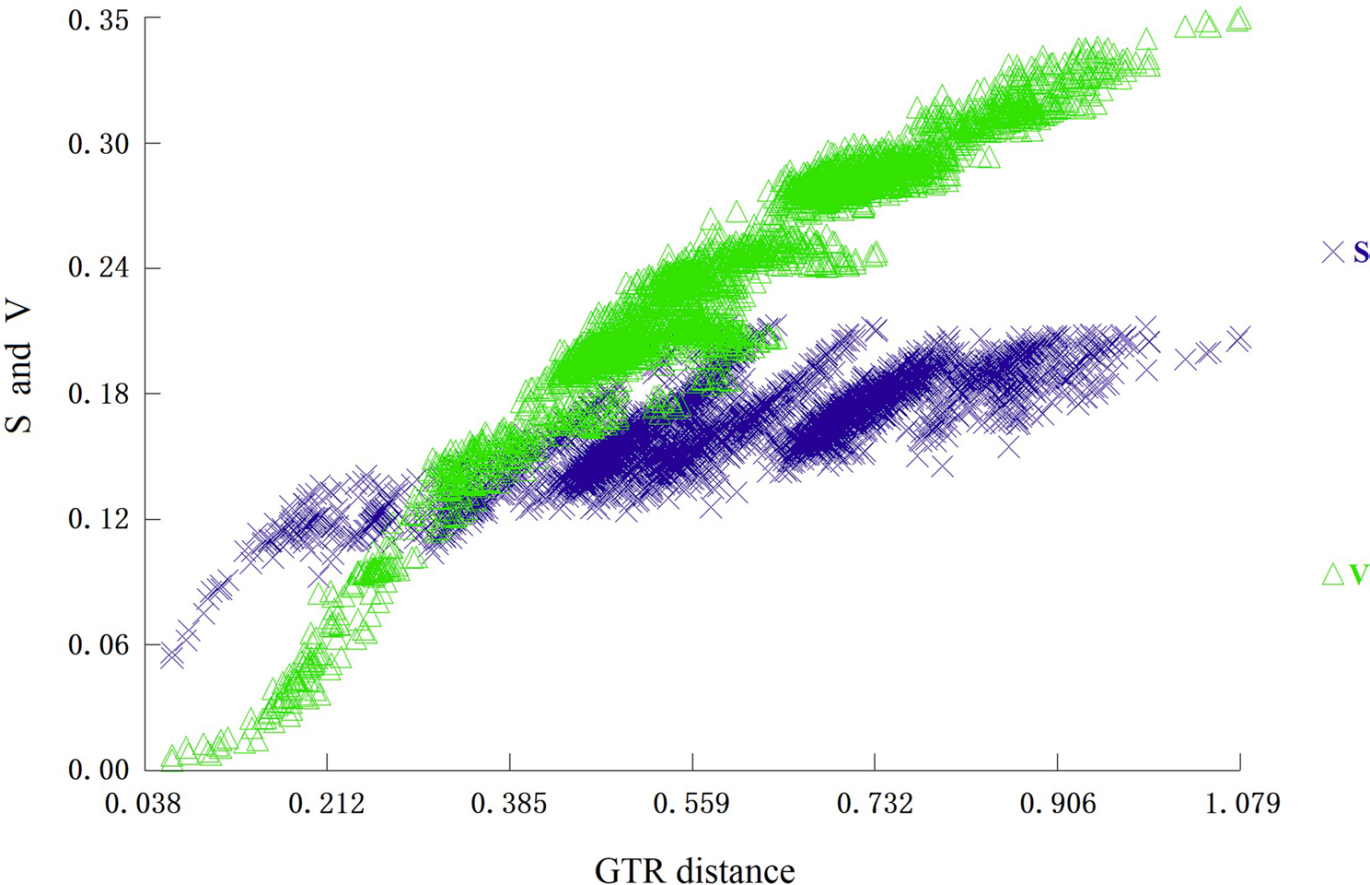
Saturation plots for 13 PCGs. The plots show the uncorrected pairwise divergence in transitions (s) and transversions (v) against the divergence calculated using the GTR model.

To investigate the evolutionary relationships among and selective pressure on 16 Neritimorpha species, we used the nonsynonymous to synonymous substitution (Ka/ Ks) ratio. The result showed that the average Ka/Ks ratio ranged from 0.060 for *cox1* to 0.766 for *nad4*. This result indicated that the 13 PCGs of all Neritimorpha mitogenomes evolved under purifying selection (Fig. 4). The Ka/Ks ratio for all PCGs was below one, indicating that the mutations yielded synonymous substitutions. The *cox1* gene has the lowest Ka/Ks ratio among studied genes and little change in amino acids; hence, it is widely used as a molecular marker for species identification and phylogenetic analysis^36,37^. The substitution saturation index value for the combined dataset of the 13 PCGs in all species (Iss = 0.685) was significantly lower than the critical values (Iss. cSym = 0.859 or Iss.cAsym = 0.847, *p* = 0.000) (Fig. 5). Thus, the combined sequence substitution was unsaturated, making the sequences suitable for phylogenetic analysis.

### Phylogenetic relationships

**Figure 6:**
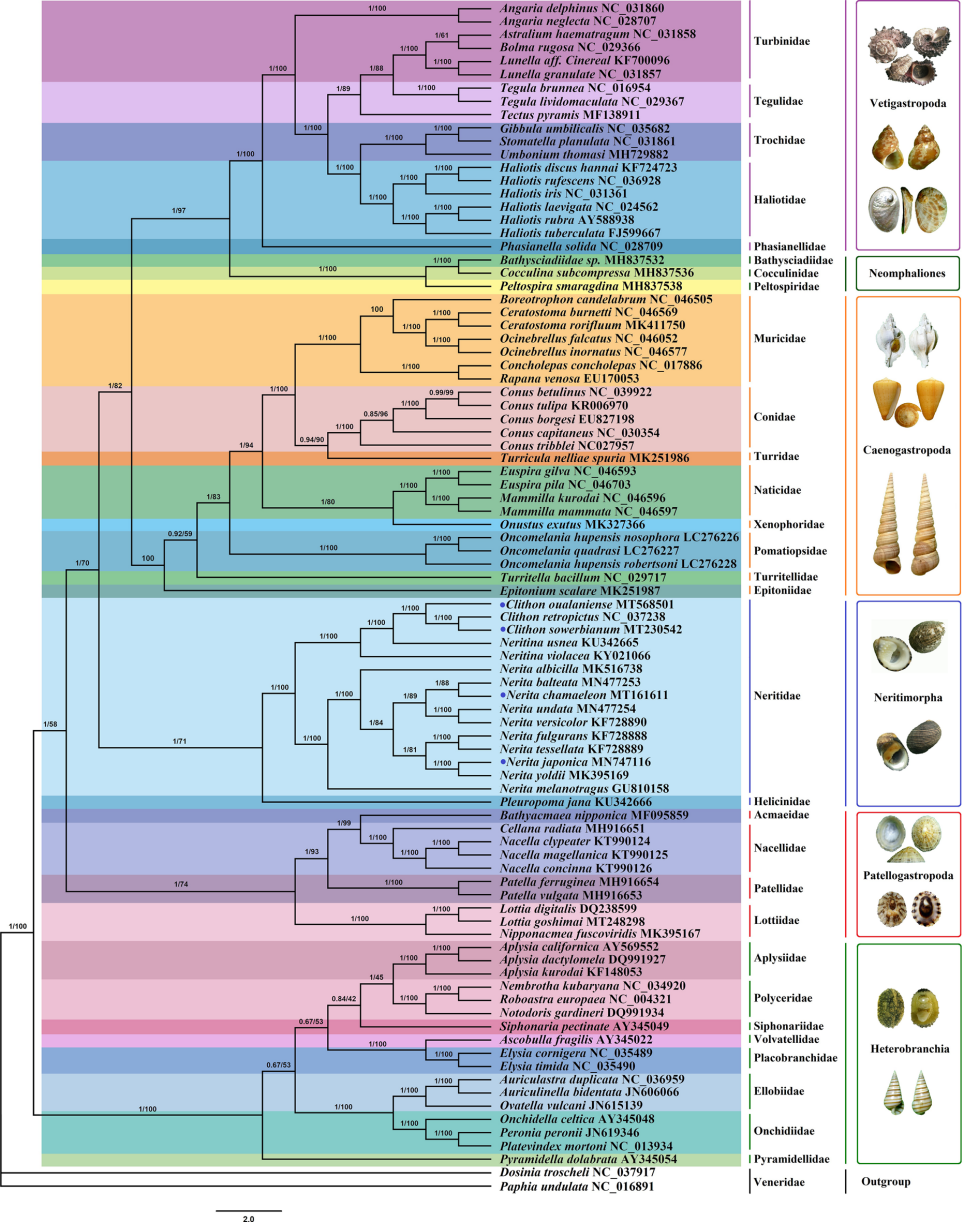
Phylogenetic tree inferred using Bayesian inference (BI) and maximum likelihood (ML) methods based on concatenated sequences of 13 PCGs from 88 gastropod mitogenomes. The sequences of two Veneridae species were chosen as the outgroups. The blue dots indicate the four Neritidae species sequenced in this study. The number at each node is the bootstrap probability.

Phylogenetic analyses were conducted on the concatenated alignment of 13 PCGs covering 88 gastropod species from thirty families of six subclasses (Vetigastropoda, Neomphaliones, Caenogastropoda, Neritimorpha, Patellogastropoda and Heterobranchia). We selected two Veneridae species (Bivalvia) as the outgroup. Maximum likelihood (ML) and Bayesian inference (BI) analyses produced almost identical topologies, with strong bootstrap and posterior probability values. However, family Lottidae of Patellogastropoda exhibited potential long-branch attraction (LBA) when we construct a Bayesian tree. Due to the large difference in branch length between members of this family and other related species, systematic errors occurred, and the true placements of these Lottidae taxa were not revealed^38,39^. This is the same as the result previously reported for the mitogenome of two limpets^40^. Finally, we combined the two methods to obtain a consistent evolutionary tree (Fig. 6).

Our phylogenetic analysis indicated that all species representing subclass Neritimorpha clustered on the same branch; meanwhile, all posterior probability values were 1, and the bootstraps values were greater than 80. Within the Gastropoda class, the six subclasses exhibited the following phylogenetic relationships: ((((Vetigastropoda + Neomphaliones) + Caenogastropoda) + Neritimorpha) + Patellogastropoda) + Heterobranchia. Neritimorpha is closely related to Caenogastropoda and Patellogastropoda. Strikingly, we found that the branching orders of Neritimorpha and Caenogastroopoda were slightly different due to the increasing abundance of Neritimorpha species.

In Neritimorpha, whole mitogenomes are available for only two families, and Helicinidae forms an independent branch. The main evolutionary pattern in the Neritimorpha was the division of Neritidae into three genera, namely, *Clithon*, *Neritina* and *Nerita*. The *Clithon* and *Neritina* species clustered together and then with the genus *Nerita*. This indicated that the genus *Clithon* has a closer genetic relationship with the genus *Neritina.* The newly sequenced species *C. sowerbianum* was the closest relative of *Clithon retropictus* and then clustered with the new experimental species *Clithon oualaniense*, followed by *Neritina usnea* and *Neritina violacea*. In the genus *Nerita*, *Nerita melanotragus* was located on a separate branch and then clustered with *Nerita albicilla*. Furthermore, two new species of the genus *Nerita*, i.e., *Nerita chamaeleon* and *Nerita japonica*, were close to *Nerita balteata* and *Nerita yoldii*, respectively*.*

### Divergence times

**Figure 7:**
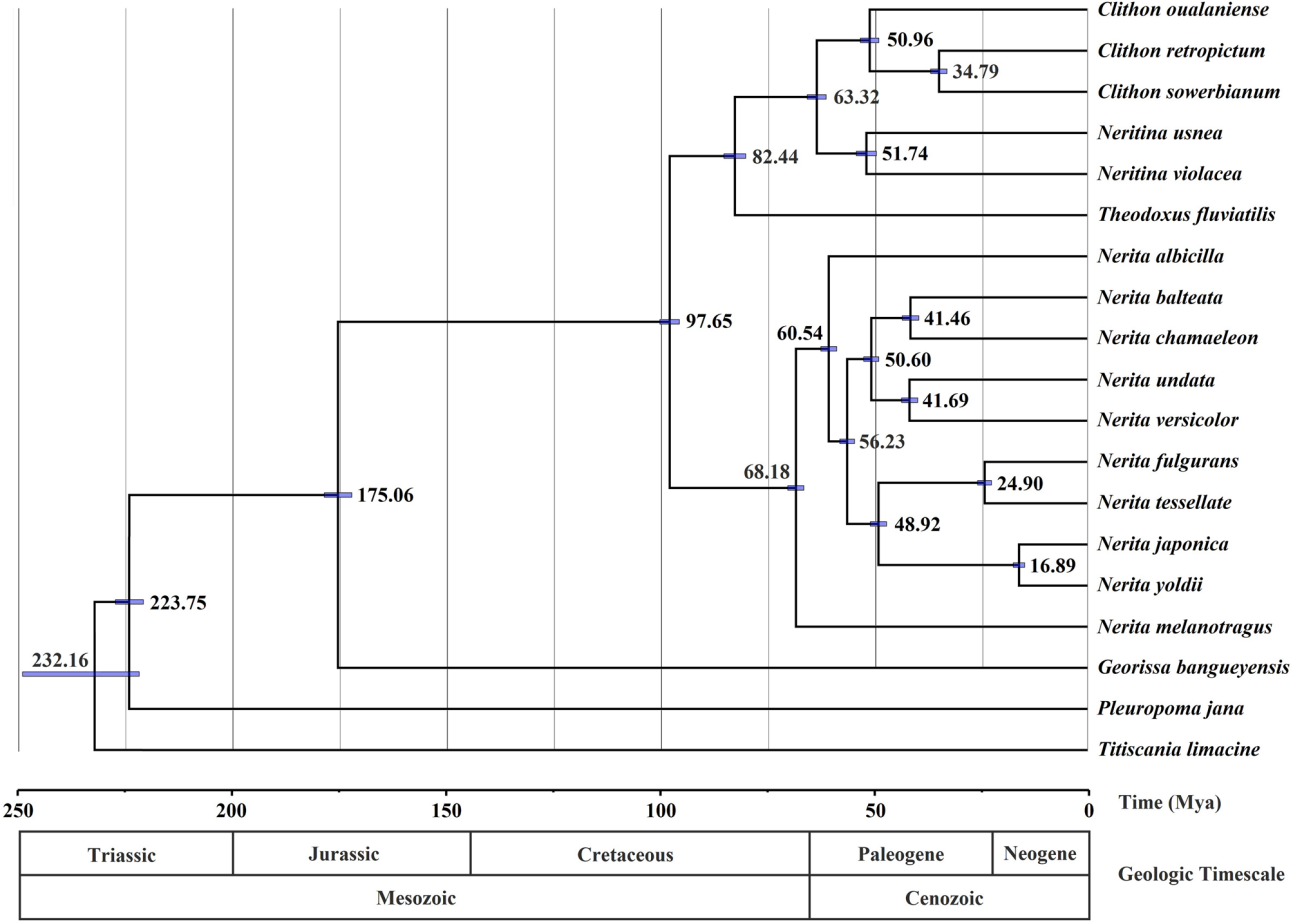
Divergence time estimation for Neritimorpha inferred via Bayesian relaxed dating methods (BEAST) based on the nucleotide sequences of 12 PCGs (excluding the *cox3* gene). Fossil samples used to calibrate internal nodes are indicated by an asterisk. The 95% HPD is reported as blue bars, and Bayesian posterior probabilities are reported for each node. The accession numbers of the sequences used in the time-calibrated tree analysis are listed in Supplementary Table S1.

The time-calibrated phylogeny indicated that Neritimorpha originated approximately 232.16 million years ago (Mya) (95% highest posterior density [HPD] interval = 268.41–231.69 Mya) (Fig. 7), in agreement with the finding of a previous study suggesting that Neritimorpha appeared in the Triassic period^30^. The Triassic was the first period of the Mesozoic, which was the transitional period of the formation of the modern biota after the disappearance of the Paleozoic biota. Great changes have taken place in marine invertebrate groups^41^. In Neritidae, the differentiation time between *Nerita* and the other three genera was the earliest (97.65 Mya). However, the estimate provided by this analysis was slightly older than the origin of the Neritidae estimated in our previous analyses (76.17–83.25 Mya)^30^. This is probably due to misidentification in the fossil record, which is determined by various taxonomic methods and influenced by different levels of experience and expertise^42^. According to our findings, especially the attribution of fossils to different genera, the fossil record of Neritidae requires a complete revision. In the genus *Nerita*, the divergence time between *N. melanotragus* and other *Nerita* species was the earliest (68.18 Mya). For years, studies on the divergence time of neritids have shown that *N. melanotragus* was the first species differentiated from *Nerita*^43^. There were 7.64 million gaps between *N. melanotragus* and *N. albicilla* and 4.31 between *N. albicilla* and other *Nerita* species.

In our study, the addition of *N. chamaeleon* and *N. japonica* changed the divergence time of *Nerita*. *N. balteata* and *N. chamaeleon* split approximately 41.46 Mya, and *N. japonica* and *N. yoldii* were differentiated approximately 16.89 Mya. Moreover, the observations for other *Nerita* species were consistent with our previous estimates of divergence time^30^. Most Neritidae species differentiation was concentrated in the Cenozoic Paleogene (approximately 2.4–65 Mya). This is the period when continental transgression was rapidly reduced and marine sediments appeared in the marginal areas of China. On the other branch, the differentiation time of *Theodoxus* species was the earliest (82.44 Mya), followed by those of *Neritina* and *Clithon* species. *N. usnea* and *N. violacea* differentiated approximately 51.74 Mya. There were 16.17 million gaps between *C. oualaniense* and the other two *Clithon* species, and *C. retropictus* and *C. sowerbianum* differentiated approximately 34.79 Mya. This geographical isolation resulting from geological movement provided environmental conditions suitable for the divergence of Neritidae, and marine sediments provided a food source for Neritidae growth.

## Conclusion

We obtained the mitogenome sequences of *C. oualaniense*, *C. sowerbianum*, *N. chamaeleon* and *N. japonica* by high-throughput sequencing, and their lengths were 15,706 bp, 15,919 bp, 15,716 bp and 15,875 bp, respectively. Each mitogenome is composed of a control region, 2 rRNAs, 13 PCGs and 22 tRNAs. The genome size, gene order and nucleotide composition of these four mitogenomes are similar to those of other neritids reported previously. Most PCGs were initiated with the ATG codon and terminated with the TAA codon. The Ka/Ks ratio indicated that these Neritimorpha species were subjected to purifying selection. Phylogenetic trees contributed to the scientific classification of Neritimorpha species. This study provides information on the genetic characteristics, phylogenetic relationships and evolution of neritids as well as a basis for resource management and selective breeding in aquaculture. These four species differentiated in the late Paleogene and early Neogene, and their evolution may be related to the geological events that changed their living environments.

## Materials and methods

### Samples and DNA extraction

Wild specimens of *C. oualaniense* (March 2020, E114°65, N22°73) were collected in the Pearl River Estuary, Guangdong Province; *C. sowerbianum* (October 2019, E110°34′, N20°08′) and *N. chamaeleon* (October 2019, E110°34′, N20°08′) were collected in Haikou, Hainan Province; and *N. japonica* (November 2018, E119°64′, N26°19′) were collected in Lianjiang, Fujian Province. All specimens were collected in the southeastern China Sea and were then preserved in absolute ethyl alcohol. The samples were identified via a published taxonomic book^44^, and we consulted taxonomists from the marine biology museum of Zhejiang Ocean University. Genomic DNA was extracted from small pieces of foot tissue taken below the operculum using the salting-out method and was stored at − 20 °C before sequencing. Only one specimen of each species was used for sequencing. All animal experiments were conducted in accordance with the guidelines and approval of the Animal Research and Ethics Committees of Zhejiang Ocean University.

### DNA sequencing and genome assembly

The mitogenomes of four Neritidae species were submitted to Origingene Bio-pharm Technology Co., Ltd. (Shanghai, China), for Illumina PE library construction and high-throughput sequencing by the Illumina HiSeq X Ten platform. Sequencing libraries with average insert sizes of approximately 400 bp were prepared. Each library generated approximately 5 Gb of raw data. Removing the low-quality and contaminated reads resulted in higher ‘N’ ratio sequences and adapters. The clean reads of the four species were de novo assembled separately using NOVOPlasty software (https://github.com/ndierckx/NOVOPlasty)^45^.

### Gene annotation and sequence analysis

Four newly assembled mitogenomes were annotated with the MITOS web server (http://mitos2.bioinf.uni-leipzig.de/index.py) based on the invertebrate genetic code^46^. Start and stop codons were confirmed using previously published Neritidae mitogenomes as references^29,30^. The circular genomes of the four Neritidae species were visualized with the CGView Server (http://stothard.afns.ualberta.ca/cgview_server/index.html)^47^. The nucleotide composition of the mitogenome for each species in family Neritidae; PCGs, tRNA genes, and rRNA genes; A and T content values; and relative synonymous codon usage (RSCU) and codon usage of PCGs were determined using MEGA 7.0^48^. The base skew values were calculated with the formulas AT skew = (A − T)/(A + T) and GC skew = (G − C)/(G + C)^49^. To test for evolutionary adaptation, rates of nonsynonymous (Ka) and synonymous (Ks) substitutions in the mitogenomes of all species of Neritidae were estimated with DnaSP 6.0^50^.

### Phylogenetic inference and divergence time estimation

Evolutionary relationships were reconstructed with the PCGs from 88 gastropod mitogenomes, the four species (*C. oualaniense*, *C. sowerbianum*, *N. chamaeleon* and *N. japonica*) newly sequenced here and two representatives of the bivalves (*Dosinia troscheli* and *Paphia undulata*) as outgroups (Table 1). Phylogenetic trees were reconstructed using BI and ML methods. The nucleotide sequences for each PCG were adjusted by DAMBE 5.3.19^51^, and substitution saturation was tested for using the GTR substitution model. Sequences for each PCG were aligned using ClustalW of MEGA 7.0^48^. Phylogenetic analyses incorporated both the maximum likelihood (ML) method using IQ-TREE^52^ and Bayesian inference (BI) using MrBayes v3.2^53^. The best-fitting model (GTR + F + R7) selected by the BIC criteria implemented in ModelFinder^54^ was used for the ML analyses. In ultrafast likelihood bootstrapping, 1000 bootstrap replicates were applied to reconstruct a consensus tree. The MrBayes settings for the best substitution model (GTR + I + G) were determined by MrModeltest 2.3^55^ under the AIC. The BI analyses involved two Markov chain Monte Carlo (MCMC) runs with 2,000,000 generations, sampling every 1000 generations and a discarded burn-in of 25%.

The estimates of divergence times among subclass Neritimorpha species were based only on nucleotide level (12 PCGs, with *cox3* excluded due to this gene being incomplete in some species) and obtained using a Bayesian framework with an uncorrelated relaxed clock and lognormal relaxed molecular clock model in BEAST v1.8.4^56^. The Yule process of speciation was used for the tree prior. For divergence time calibration, two calibration points were used as the prior for the corresponding split divergence time. Priors for fossil ages were drawn from normal distributions, and the root *Pleuropoma jana* was constrained between 235 and 223 million years ago (MYA)^57^. The 80 Ma point calibration was set as the root rate of *Nerita* based on the fossil of *Nerita melanotragus* (95–80 MYA)^58^. The final Markov chain was run twice for 100 million generations, with sampling every 1000 generations and 10% of samples discarded as a burn-in by TreeAnnotator v1.8.4 software (in the BEAST package). Then, using Tracer v. 1.6^59^, chain convergence was confirmed, and the majority of the values exceeded an effective sample size (ESSs) of 200. The phylogenetic tree and divergence times were visualized using FigTree v1.4.3 software^60^.

## Supplementary Information

Supplementary Information.

## Data Availability

The mitochondrial genome data has been submitted to NCBI GenBank under the following accession numbers: *Clithon oualaniense* (MT568501), *Clithon sowerbianum* (MT230542), *Nerita chamaeleon* (MT161611), *Nerita japonica* (MN747116).
