# Supplementary material for: Characterization of four mitochondrial genomes of family Neritidae (Gastropoda: Neritimorpha) and insight into its phylogenetic relationships
Source: Sci Rep. 2021 Jun 3;11:11748. doi: 10.1038/s41598-021-91313-0 (PMC8175686; doi:10.1038/s41598-021-91313-0)
Supplement: Supplementary file 1 — Supplementary Information. [file 41598_2021_91313_MOESM1_ESM.docx]

| **Superfamily** | **Family** | **Genus** | **Species** | **Accession no.** |
| --- | --- | --- | --- | --- |
| Neritoidea | Neritidae | *Clithon* | *Clithon oualaniense* | MT568501 |
|  |  |  | *Clithon retropictus* | NC_037238 |
|  |  |  | *Clithon sowerbianum* | MT230542 |
|  |  | *Neritina* | *Neritina usnea* | KU342665 |
|  |  |  | *Neritina violacea* | KY021066 |
|  |  | *Theodoxus* | *Theodoxus fluviatilis* | KU342667 |
|  |  | *Nerita* | *Nerita albicilla* | MK516738 |
|  |  |  | *Nerita balteata* | MN477253 |
|  |  |  | *Nerita chamaeleon* | MT161611 |
|  |  |  | *Nerita undata* | MN477254 |
|  |  |  | *Nerita versicolor* | KF728890 |
|  |  |  | *Nerita fulgurans* | KF728888 |
|  |  |  | *Nerita tessellata* | KF728889 |
|  |  |  | *Nerita japonica* | MN747116 |
|  |  |  | *Nerita yoldii* | MK395169 |
|  |  |  | *Nerita melanotragus* | GU810158 |
| Hydrocenoidea | Hydrocenidae | *Georissa* | *Georissa bangueyensis* | KU342664 |
| Helicinoidea | Helicinidae | *Pleuropoma* | *Pleuropoma jana* | KU342666 |
| Neritopsoidea | Neritopsidae | *Titiscania* | *Titiscania limacine* | KU342669 |

**Table S1.** The accession numbers of the nineteen Neritimorpha sequences used in the time-calibrated tree analysis.
